# Does environmental regulation lessen health risks? Evidence from Chinese cities

**DOI:** 10.3389/fpubh.2023.1322666

**Published:** 2024-01-11

**Authors:** Qingqing Xu, Liyun Wang, Hanxue Hou, ZhengChang Han, Wenhao Xue

**Affiliations:** ^1^School of Economics, Qingdao University, Qingdao, China; ^2^ShanDong ZhengYuan Geophysical Information Technology Co., Ltd., Jinan, China

**Keywords:** environmental regulation, integrated exposure-response model, health risk, two-way fixed effects model, PM_2.5_

## Abstract

**Introduction:**

Atmospheric pollution is a severe problem confronting the world today, endangering not only natural ecosystem equilibrium but also human life and health. As a result, governments have enacted environmental regulations to minimize pollutant emissions, enhance air quality and protect public health. In this setting, it is critical to explore the health implications of environmental regulation.

**Methods:**

Based on city panel data from 2009 to 2020, the influence of environmental regulatory intensity on health risks in China is examined in this study.

**Results:**

It is discovered that enhanced environmental regulation significantly reduces health risks in cities, with each 1-unit increase in the degree of environmental regulation lowering the total number of local premature deaths from stroke, ischemic heart disease, and lung cancer by approximately 15.4%, a finding that remains true after multiple robustness tests. Furthermore, advances in science and technology are shown to boost the health benefits from environmental regulation. We also discover that inland cities, southern cities, and non-low-carbon pilot cities benefit more from environmental regulation.

**Discussion:**

The results of this research can serve as a theoretical and empirical foundation for comprehending the social welfare consequences of environmental regulation and for guiding environmental regulation decision-making.

## 1 Introduction

China has implemented a development strategy dominated by heavy industry since the initiation of reform and opening. This strategy has contributed to rapid economic expansion but has also been accompanied by several challenges. Particularly the large amount of pollutants emitted by industrial activities seriously harmed air quality and risked people’s health and well-being (1, 2). According to the Report on the State of the Ecology and Environment in China 2020, 135 of China’s 337 cities had substandard air quality in 2020, with these numbers accounting for 40% of the total number of cities. Such poor environmental conditions have caused economic losses in China ranging from 8 to 15% of GDP and have jeopardized people’s health rights and interests. Residents’ long-term exposure to high levels of fine particulate matter (PM_2.5_) has been found to increase the incidence of stroke, ischemic heart disease, and lung cancer in epidemiological studies (3–5). As a result, the conflict between air pollution and residents’ health is becoming increasingly visible, and it has become a practical concern that cannot be disregarded in China’s growth. Additionally, enhancing environmental governance to alleviate air pollution has emerged as a widely shared concern. Consequently, the Chinese government has introduced several regulations and practices to control and supervise the pollution-emitting behaviors of enterprises and individuals to alleviate the air pollution issue. For example, the Law on the Prevention and Control of Atmospheric Pollution was enacted by the Chinese government in 1987 to provide a legal basis for environmental regulation (6), followed by the Cleaner Production Promotion Law in 2003 to enhance the environmental friendliness of industrial production (7). In 2006, the Chinese government broke down the emission reduction targets for the provincial administrative regions, achieving the shift from concentration control to total pollutant amount control. As a result, environmental regulation transitioned from “soft constraints” to “hard constraints” (8), with the adoption of administrative orders or government performance assessment.

The concept of “regulation” was introduced by the American economist Kahn in his book The Economics of Regulation: Principles and Institutions (9), where he defined it as an institutional arrangement that substitutes government directives for market competition to achieve good economic performance. With the growing environmental problems resulting from the crude economic model, environmental regulation has become a significant branch of regulatory economics (10). Environmental regulation refers to the government’s direct or indirect control and management of pollution sources to improve the quality of the ecological environment (11). The intensity of environmental regulation is often indirectly measured by some alternative indicators, such as industrial emissions (12), pollution control investment (13), and operating costs (14). These indicators have facilitated research on environmental regulation in the area of air pollution.

The function of environmental regulation in decreasing pollution has been well supported by evidence, and various viewpoints and levels have been adopted to examine the influence of environmental regulations and policies on pollution emissions in China. For example, Du and Li (15), Feng et al. (16), and Zhang et al. (17) analyzed the emissions of CO_2_ and PM_2.5_ from industrial businesses, cities, and regions, respectively, and discovered that environmental regulations can effectively lower these pollutants, especially in the eastern, central, and northeastern areas. Yu et al. (18) assessed the impact of the Air Pollution Prevention and Control Action Plan on the emissions of PM_2.5_ and SO_2_ from Chinese cities, and the results indicated that these policies can significantly improve air quality. Using a time-varying difference-in-difference (DID) model, Liu et al. (19) demonstrated that China’s low-carbon city pilot policy effectively reduced CO_2_ emissions in the pilot cities, but the effects varied across regions and administrative levels. Hu et al. (20) found that environmental protection taxes can significantly reduce air pollutants such as PM_2.5_, SO_2_, NOx, and CO in areas with large economic and industrial sectors with high emission intensities in the short term, as well as offering substantial co-benefits in global climate change mitigation. Meanwhile, the health benefits of environmental regulation have attracted scholarly attention as regulatory intensity has increased. The majority of the researchers argue that environmental regulation reduces health risks and mortality rates. For instance, a study conducted in China revealed that the infant mortality rates in the “two control zones” with stringent environmental regulation declined by 20% compared to other zones (21). Similarly, a study in the United States demonstrated that the shutdown of coal-fired power plants significantly decreased the percentage of low-birth-weight and preterm infants by 15 and 28 percent, respectively, in the downwind states (22). Employing the difference-in-differences (DID) model, Xu et al. (23) examined the impact of the Eleventh Five-Year Plan’s environmental regulations on human health, discovering that environmental regulations reduce the risk of injury or illness among adults by 9.2 percent, with this effect being more pronounced among males, rural residents, and low-income households. Zhou et al. (24) also demonstrated that more stringent environmental regulations for businesses benefit public health. Similarly, Zhang et al. (25) examined the influence of atmospheric environmental policy on public health, demonstrating that it decreases the intensity of soot emissions and mitigates the detrimental health effects of air pollution. However, other research has implied that there is a critical value between environmental regulation and public health rather than a simple linear link: if the critical value is exceeded, environmental regulation may lead to negative economic and social consequences, such as lowering economic growth, increasing unemployment and poverty, widening the urban–rural income gap, and undermining economic efficiency, all of which have an indirect negative impact on population health (26).

Although, as mentioned earlier, studies have been conducted to examine the influence of environmental regulation on health, they are still limited and are still at the exploratory stage, with few studies probing the underlying mechanisms. Additionally, the impact of regional heterogeneity factors, such as geographic location and environmental patterns, on the health benefits of environmental regulation has tended to be overlooked in previous studies. For example, cities in different geographical locations may suffer from different levels and types of air pollution problems, necessitating different intensities and forms of environmental regulatory policies; similarly, cities with diverse environmental patterns may have varying levels of air quality and environmental governance. These characteristics of regional heterogeneity may affect the applicability and effectiveness of environmental regulation, leading to different health benefits. For this reason, by utilizing data on stroke (STK), ischemic heart disease (IHD), and lung cancer (LC) as the health endpoints for PM_2.5_ health risk assessment, this study explores the effect of environmental regulation on health risk through a two-way fixed effects model. Next, the moderating role of technological innovation in the health benefits of environmental regulation is explored. Moreover, we investigate the implications of city heterogeneity characteristics on the health advantages of environmental regulation by analyzing factors based on regional location and environmental differences. The findings of this study can provide useful scientific evidence and an important frame of reference for the formulation of more strategic and sound environmental regulatory policies and for the enhancement of the health benefits stemming from environmental regulation. This study thus has both theoretical and practical relevance.

The rest of this paper is structured as follows: The study area of the research is presented in Section 2. Section 3 discusses the data sources, variable selection, and model development. Section 4 reports the results of the benchmark regression, instrumental variable model, robustness, mechanism effects, and heterogeneity analyses, as well as their interpretation and analysis. Section 5 summarizes the findings, and policy recommendations are presented therein.

## 2 Materials and methods

### 2.1 Study area

**Figure 1:**
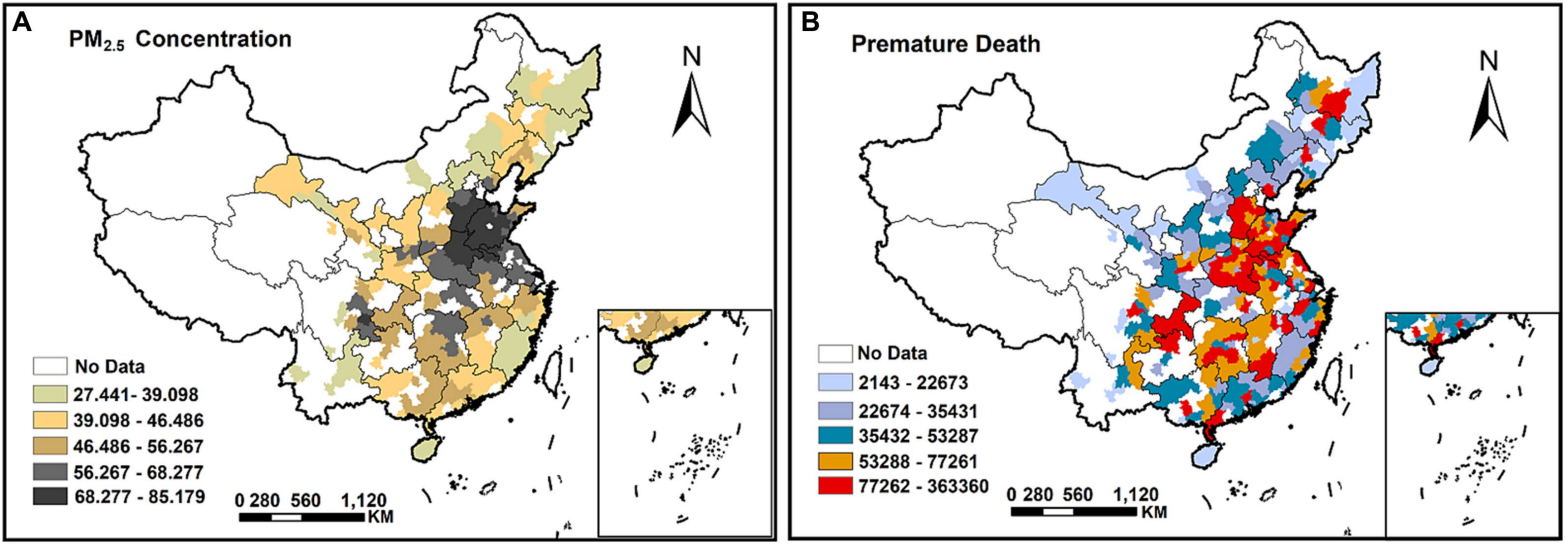
The spatial distribution in China for PM_2.5_ concentrations **(A)** and the number of premature deaths (estimated by the integrated exposure-response model) from STK, IHD, and LC **(B)**.

There are 279 cities in mainland China included in the study area, and the study duration spans the years 2009–2020. Figure 1 depicts the study area’s spatial distribution, with (a) the PM_2.5_ concentration levels in each Chinese city and (b) the number of premature deaths from the three diseases of STK, IHD, and LC estimated by the integrated exposure-response model.

### 2.2 Data and variable selections

#### 2.2.1 Explained variable

In this paper, three common attributable deaths from circulatory and respiratory diseases were chosen as health endpoints, e.g., stroke (STK, International Classification of Diseases Revision 10 code/ICD-10: 160–169), ischemic heart disease (IHD, ICD-10: 120–125), and lung cancer (LC, ICD-10: C33–C34). According to Dai et al. (27) and Burnett et al. (28), the relative mortality risk of these three health endpoints due to PM_2.5_ exposure was calculated using the integrated exposure-response (IER) model (Equations 1–3), and the corresponding number of premature deaths was estimated and used as the explanatory variables in this study. The specific algorithm is as follows:

Step 1: Calculation of relative mortality risk


(1) $$RR_{i}\left(K\right)=\{\begin{array}{l} \;1,\;K\leq K_{0}\\ 1+\alpha \left\{1-\exp\left[-\gamma {\left(K-K_{0}\right)}^{\delta }\right]\right\},K>K_{0} \end{array}$$

The relative health risk of PM_2.5_-producing disease i(i=1, 2, 3) at concentration K is denoted by ${\text{RR}}_{i}\left(K\right)$.$\;K_{0}$ indicates the health effect threshold; when the PM_2.5_ concentration is below $K_{0}$, there is no negative health effect and ${\text{RR}}_{i}\left(K\right)$=1; however, when the concentration crosses the threshold, the relative risk increases with increasing concentration, with $RR_{i}\left(K\right)=1+\alpha \left\{1-\exp\left[-\gamma {\left(K-K_{0}\right)}^{\delta }\right]\right\}$. The values of the parameters in Equation (1) are shown in Table 1 and are based on the research of Lee et al. (29).

**Table 1:** Coefficients in model [Equation (1)].

	STK	IHD	LC
K_0_ (μg/m^3^)	8.38	6.96	7.24
α	1.01	0.843	159
γ	0.0164	0.0724	0.000119
δ	1.14	0.544	0.735

Step 2: Estimation of the number of premature deaths.


(2) $${\text{ED}}_{i}=\left(1-1/{\text{RR}}_{i}\right)\times D_{i}\times P$$

(3) $$\text{ED}=\sum_{i=1}^{3}{\text{ED}}_{i}$$

where ${\text{ED}}_{i}$ denotes the premature deaths from disease i induced by outdoor PM_2.5_ exposure, ED indicates the total number of premature deaths from STK, IHD, and LC, *P* is the number of people exposed to a given pollution concentration, and $D_{i}$ refers to the baseline mortality rate for each disease for the year.

The PM_2.5_ concentration data employed in this paper were obtained from the China High Air Pollutants (CHAP) database^1^ (30, 31) and were collected using satellite remote sensing and machine learning technologies at high temporal and spatial resolutions of 1 day and 1 kilometer, respectively. The baseline mortality rate was derived from the National Bureau of Statistics of China data, with mortality rates for various disease categories being counted in both urban and rural areas. For this reason, the population-wide baseline mortality rates for each disease from 2009 to 2020 were calculated by employing the statistical ratios of the urban and rural populations to the total population as weights (32). The ED was logarithmized in this study to eliminate the disturbance of heteroskedasticity. The descriptive statistics for the selected variables in the model are displayed in Table 2. During the period 2009–2020, the average number of premature deaths from the three diseases that were induced by outdoor PM_2.5_ exposure in China was 50,004, with a maximum value of 413,864. These numbers indicates that air pollution and related health risks are still quite serious in China.

**Table 2:** The results of descriptive statistics.

Variable	Unit	*N*	Mean	S. D.	Min	Max
ED	People	3,316	50,004	38,002	1,651	413,864
*ER*	–	3,336	0.191	0.068	0.046	0.875
*RGDP*	10^4^CNY	3,347	1.380	0.892	0.004	14.520
*PD*	People/km^2^	3,348	444.460	344.701	4.971	3239.860
*TIV*	%	3,348	41.058	10.083	14.360	83.870
*FSR*	%	3,344	0.457	0.224	0.041	1.541
*NH*	–	3,329	185.441	178.006	8.000	3052.000
*AR*	MJ/m^2^	3,348	12.333	1.179	8.396	16.235
*AN*	–	3,069	0.499	0.136	0.059	0.780
*AP*	m	3,348	0.003	0.002	<0.001	0.008
*AT*	k	3,348	287.7	5.327	273.9	299.1

#### 2.2.2 Explanatory variable

Environmental regulation intensity (ER) is the explanatory variable in this research. Three indicators reflecting the level of environmental protection were chosen to capture the environmental regulation intensity of different cities (33), e.g., the comprehensive utilization rate of industrial solid waste, the centralized sewage treatment rate, and the rate of harmless treatment of domestic garbage (34). Specifically, the comprehensive utilization rate of industrial solid waste is calculated as the ratio of the comprehensively utilized industrial solid waste to the sum of the generated industrial solid waste and the comprehensively utilized storage in previous years. The rate of centralized sewage treatment is determined as the ratio of the wastewater discharged that uses centralized wastewater treatment to the total wastewater discharged. The rate of harmless treatment of domestic waste is stated as the ratio of the domestic garbage that is harmlessly disposed of to the domestic garbage created.

The entropy weighting technique, a method that can eliminate the involvement of subjective factors, was employed to identify the weights of each indicator. Then, the indicators were multiplied by their corresponding weights after normalization, and the resulting composite environmental regulatory intensity index was calculated. This composite index can reflect the degree of importance attached to environmental problems and the effectiveness of response measures in different regions. Namely, the higher the index value is, the greater the environmental regulation intensity and the importance attached to environmental protection. Data for the environmental regulation intensity are gathered from the China City Statistical Yearbook.

#### 2.2.3 Control variable

In this research, control variables for both socioeconomic factors and natural elements are incorporated into the empirical analysis. Control variables for socioeconomic characteristics include real GDP *per capita* (*RGDP*), population density (*PD*), tertiary industry value added as a share of GDP (*TIV*), financial self-sufficiency rate (*FSR*), and number of hospitals and health centers (*NH*) (35, 36), with these variables being based on data from the China Urban Statistical Yearbook (CUSY). For natural components, the control variables include annual radiation (*AR*), annual normalized difference vegetation index (*AN*), annual precipitation (*AP*), and annual temperature (*AT*) (37, 38), with data gathered from the European Center for Medium-Range Weather Forecasts (ECMWF) ERA5 dataset (39). The variables are addressed in light of previous studies as follows: Real GDP *per capita* is calculated with deflator-adjusted GDP *per capita* (i.e., the ratio of nominal GDP to real GDP), thus eliminating the effect of the inflation factor (40). The fiscal self-sufficiency rate is expressed in terms of the proportion of local budget revenues to expenditures (41). The missing data are filled in by interpolation. Logarithmic transformation is used for processing non-ratio-type data to limit heteroskedasticity and ensure the uniformity of variables in the order of magnitude. Following the aforementioned processing, this study compiles a robust collection of fundamental data for empirical investigation.

#### 2.2.4 Mechanism variable

The degree of science and technology is incorporated into the study framework to investigate the potential factors that influence the health advantages of environmental regulation. Specifically, the level of scientific and technological development (*TE*), expressed as the ratio of fiscal expenditure for science to fiscal revenue (42), reflects a region’s capacity for scientific and technological innovation as well as the degree of scientific and technological support for environmental governance, with higher ratios suggesting higher investment in scientific and technological development and a greater contribution of science and technology to environmental governance. The information for this variable was obtained from the China Urban Statistical Yearbook.

### 2.3 Methodology

#### 2.3.1 Benchmark regression model

**Figure 2:**
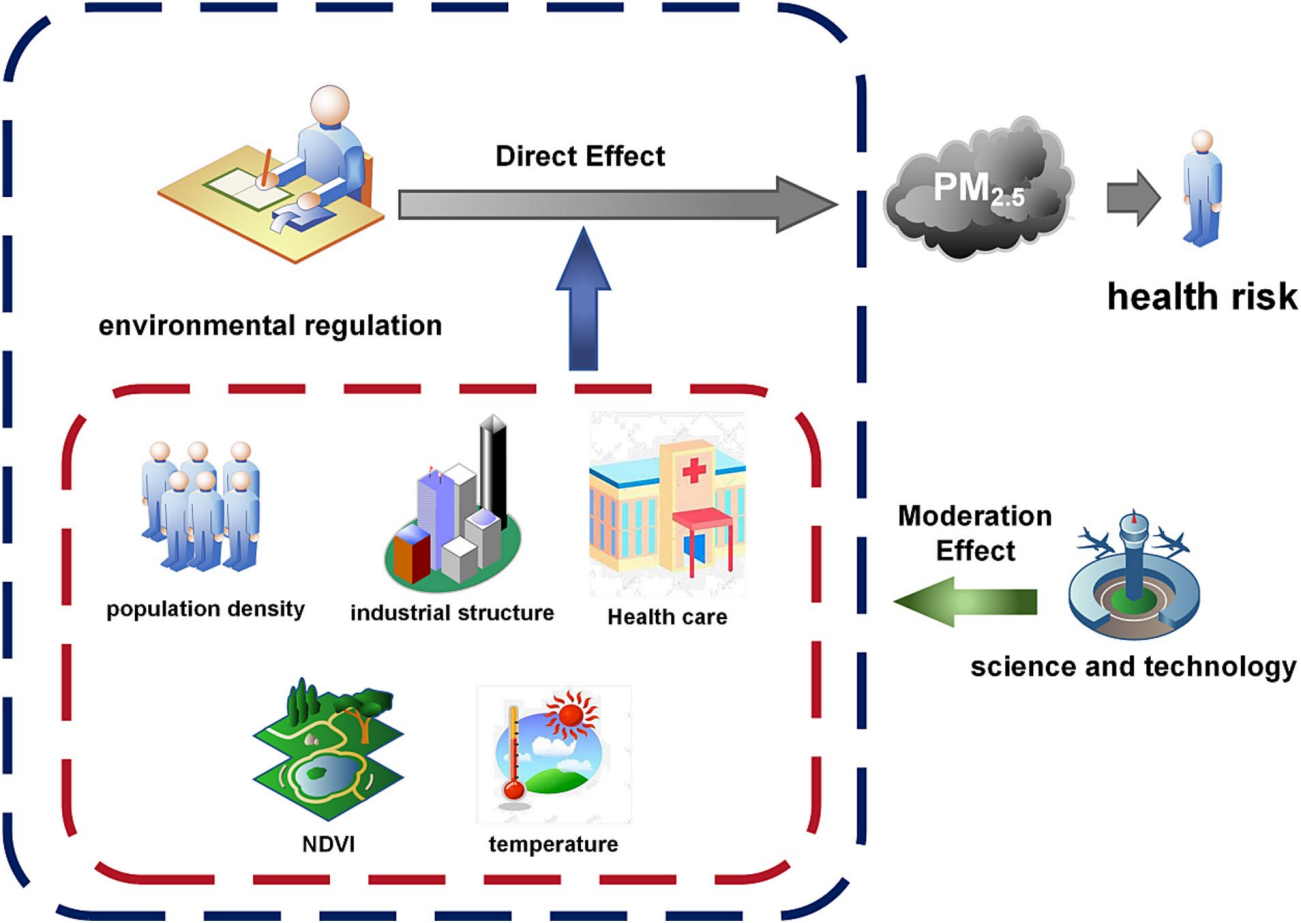
The overall process structure of the empirical model.

In this research, a two-way fixed effects model is constructed to evaluate the health implications of environmental regulation, with the particular model established as follows:


(4) $$D_{it}=\alpha +\beta ER_{it}+\gamma X_{it}+\vartheta_{i}+\varphi_{t}+\varepsilon_{it}$$

where $D_{\text{it}}$ denotes the total number of STK, IHD, and LC premature deaths induced by outdoor PM_2.5_ exposure in city i in year t. ${\text{ER}}_{\text{it}}$ is the environmental regulation intensity index in city i in year t. $X_{\text{it}}$ represents the set of control variables. The city-and time-fixed effects are denoted by $\vartheta_{i}$ and $\varphi_{t}$, respectively, and the random error term is denoted by $\varepsilon_{\text{it}}$. The coefficient β is the focus of this research, responding to the implications of environmental regulation on health risk (number of premature deaths). Additionally, the flowchart of the empirical exploration for this study is depicted in Figure 2.

#### 2.3.2 Mechanism analysis

To examine the role of science and technology as a mechanism variable, we integrated the interaction term between science and technology level (*TE*) and the environmental regulation intensity index (*ER*) in the analytical model and developed Equation (5) for regression:


(5) $$D_{\text{it}}=\alpha +\beta {\text{ER}}_{it}+\rho {\text{TE}}_{\text{it}}\times {\text{ER}}_{\text{it}}+\gamma X_{\text{it}}+\vartheta_{i}+\varphi_{t}+\varepsilon_{\text{it}}$$

where ${\text{TE}}_{\text{it}}$ represents the mechanism variable, i.e., the level of science and technology, and the remaining variables are identical to those in Equation (4). The statistical significance of the coefficient ρ was examined to evaluate the influence of science and technology on the health outcomes of environmental regulation. As a result, the significance of *TE* as a moderating variable will be captured here.

## 3 Results and discussion

### 3.1 Results of descriptive analysis

As shown in Figure 1, areas of heavy PM_2.5_ pollution and areas of high premature deaths have a high level of spatial overlap, primarily in economically developed areas such as the North China Plain and the Yangtze River Delta. Notably, the higher the PM_2.5_ concentration is, the greater the number of premature deaths, with a clear positive correlation between the two.

### 3.2 Overall effect

Table 3 illustrates the impact of environmental regulations on health risk. Column (1) adjusts for city-and time-fixed effects. Column (2) incorporates control variables for economic and social aspects. Column (3) adds extra control variables for natural factors. It can be seen that the *ER* coefficients are negative and statistically significant (*p* < 0.05) in all models (Columns 1–3), demonstrating that increased environmental regulation intensity effectively decreases health risks. In particular, the coefficient of *ER* is-0.154 (*p* < 0.05) in the most refined model (Column 3), and there is no substantial variations in the size or significance level of the coefficient when this model is compared to the other two models, indicating that the results showing the adverse impact of environmental regulation on health risk are robust. The increased intensity of environmental regulation has raised the environmental awareness and behavior of enterprises and residents. This encourages them to adopt more energy-efficient and emission-reducing production methods as well as more environmentally friendly lifestyles, thus lowering the level of industrial pollutant emissions and pollution from anthropogenic activities (43, 44); furthermore, it has also facilitated the development and application of green technologies, such as clean energy (45), resulting in lower energy consumption. All these factors contribute to lower air pollutants such as PM_2.5_, improving air quality, and reducing the risk of disease and death.

**Table 3:** The results of the baseline regression.

	(1)	(2)	(3)
	ED	ED	ED
*ER*	−0.296^***^	−0.273^***^	−0.154^**^
	(−3.18)	(−3.41)	(−2.34)
*RGDP*		−0.043^***^	−0.005
		(−4.04)	(−0.65)
*LnPD*		0.707^***^	0.364^**^
		(3.44)	(1.99)
*TIV*		−0.003^***^	−0.001^**^
		(−3.34)	(−2.36)
*FSR*		−0.075^**^	−0.043
		(−2.11)	(−1.52)
*LnNH*		0.066^***^	0.041^***^
		(5.77)	(4.46)
*LnAR*			−0.025
			(−0.37)
*LnAN*			−0.722^***^
			(−5.07)
*LnAP*			2.274
			(0.69)
*LnAT*			4.922^***^
			(3.73)
_cons	10.621^***^	6.403^***^	−19.156^**^
	(595.00)	(5.41)	(−2.53)
City FE	Yes	Yes	Yes
Time FE	Yes	Yes	Yes
N	3,316	3,313	3,035
*R* ^2^	0.982	0.985	0.993

t statistics in parentheses; ^*^p < 0.10, ^**^p < 0.05, ^***^p < 0.01.

Moreover, there are some noticeable outcomes from the control variables. Population density and the number of hospitals and health centers are both positively associated with health risks, with coefficients of 0.364 (*p* < 0.05) and 0.041 (*p* < 0.01), respectively. In contrast, the value added for tertiary industry as a proportion of GDP was adversely associated with health risk, with a coefficient of −0.001 (*p* < 0.05). This is because human activities unavoidably degrade the environment as the population density of a city rises, which in turn leads to an increase in the risk of disease for residents (46). The number of hospitals and health centers in a city reflects the level of medical resources and the capacity of public health services; however, there is a positive correlation between the number of hospitals and health risks, which may indicate that there is a high demand for medical services among the population, but the distribution of urban health care resources is unbalanced, which leads to a poorer health in some areas or populations. In addition, the service-led tertiary industry is less damaging to the environment than the secondary industry, and the higher the proportion of the tertiary industry’s value added in GDP, the more it indicates that the industrial structure has been optimized and industrial pollution emissions have been relatively reduced, resulting in a reduction in health risks. For natural factors, there is a promotive association between annual temperature and health risk with a coefficient of 4.922 (*p* < 0.01). Annual normalized difference vegetation index (NDVI), in contrast, was shown to be adversely connected with health risk with a coefficient of-0.722 (*p* < 0.01). The reason for this is that a higher NDVI indicates greater vegetation cover, which can capture and immobilize atmospheric particles such as PM_2.5_ and PM_10_ via structures such as sticky substances and capillaries on the surface of leaves, reducing the concentration of these particles in the air and thus effectively lowering the human health risk caused by outdoor PM_2.5_ pollution (47).

### 3.3 Robustness check

#### 3.3.1 Endogenous treatment

The composite index of environmental regulation lagged by one period is employed as an instrumental variable for two-stage least squares (2SLS) estimation to address the potential endogeneity due to missing variables. This model has an *F* value of 385.88 in the first-stage regression, which is higher than 10 and passes the significance test at the 1% level, demonstrating that there is no issue of a weak instrumental variable (48). Additionally, the estimation results of the second stage are shown in Column (1) of Table 4. These results reveal that environmental regulation exerts a significant negative influence on health risk, and that the total number of premature deaths in STK, IHD, and LC caused by outdoor PM_2.5_ pollution will be reduced by 14.7% with a 1-unit increase in the level of environmental regulation, suggesting that, after accounting for probable endogeneity, the dampening consequence of increasing the intensity of environmental regulation on health risks remains significant, which is aligned with the findings of the preceding analysis.

**Table 4:** The results of the robustness test.

	(1)	(2)	(3)	(4)	(5)
	ED	ED	ED	ED	ED
*ER*1		−0.154^**^			
		(−2.34)			
*ER*	−0.147^**^		−0.155^**^	−0.208^***^	−0.123^*^
	(−2.14)		(−2.36)	(−2.78)	(−1.92)
*RGDP*	−0.005	−0.005	−0.005	−0.009	−0.006
	(−0.68)	(−0.65)	(−0.61)	(−1.39)	(−0.98)
*LnPD*	0.386^**^	0.364^**^	0.363^**^	0.280	0.285
	(2.12)	(1.99)	(1.99)	(1.48)	(1.54)
*TIV*	−0.001^**^	−0.001^**^	−0.001^**^	−0.001^**^	−0.001
	(−2.46)	(−2.36)	(−2.39)	(−2.20)	(−1.62)
*FSR*	−0.037	−0.043	−0.044	−0.043	−0.026
	(−1.31)	(−1.52)	(−1.54)	(−1.41)	(−0.95)
*LnNH*	0.039^***^	0.041^***^	0.041^***^	0.048^***^	0.033^***^
	(4.38)	(4.46)	(4.46)	(4.79)	(3.77)
*LnAR*	−0.016	−0.025	−0.029	−0.005	−0.013
	(−0.23)	(−0.37)	(−0.43)	(−0.08)	(−0.20)
*LnAN*	−0.664^***^	−0.722^***^	−0.713^***^	−0.732^***^	−0.491^***^
	(−4.81)	(−5.07)	(−4.95)	(−5.20)	(−3.54)
*LnAP*	3.009	2.274	2.032	3.420	8.471^***^
	(0.84)	(0.69)	(0.62)	(0.99)	(2.92)
*LnAT*	5.158^***^	4.922^***^	4.940^***^	3.790^***^	4.855^***^
	(4.03)	(3.73)	(3.69)	(2.87)	(3.55)
_cons	−19.736^***^	−19.156^**^	−19.260^**^	−12.328	−18.437^**^
	(−2.72)	(−2.53)	(−2.51)	(−1.63)	(−2.43)
City FE	Yes	Yes	Yes	Yes	Yes
Time FE	Yes	Yes	Yes	Yes	Yes
*N*	2,759	3,035	2,991	3,035	2,756
*R* ^2^	0.993	0.993	0.993	0.993	0.994

t statistics in parentheses; ^*^p < 0.10, ^**^p < 0.05, ^***^p < 0.01.

#### 3.3.2 Replacement of explanatory variable measures

The entropy weight technique is employed in the baseline regression model to obtain a composite index of environmental regulation intensity. To confirm the validity of the estimates, the environmental regulation composite index (*ER1*) is also calculated using the equal-weight approach and replaces the original explanatory variables with it to conduct another regression analysis. The regression results after replacing the explanatory variable measurement method are available in Column (2) of Table 4. The coefficient of *ER1* is-0.154 (*p* < 0.05), demonstrating that the effect of the environmental regulation composite index on health risk, whether calculated by entropy or equal weighting, is negative and significant with a similar coefficient magnitude, thus confirming the robustness of the baseline regression outcomes of this study.

#### 3.3.3 Excluding data from four municipalities

Given the large differences in administrative levels and socioeconomic development environments between the four municipalities (Beijing, Chongqing, Shanghai, and Tianjin) and other cities, these four municipalities are separated from the full sample in this study and then the regression analyses are carried out again to exclude the possible influence of these factors on the benchmark regression findings (49). Column (3) of Table 4 displays the regression results after removing the data from the four municipalities. Notably, these results are not appreciably distinct from the results of the baseline regression, with the coefficient of *ER* being-0.155 (*p* < 0.05), confirming that the core findings of the research are reliable.

#### 3.3.4 Excluding the interference of outliers

This study winsorizes the extreme values of continuous variables to weaken the impact of outliers on the empirical analysis by replacing the values at the 1 and 99% quartiles with the corresponding truncated values, which preserves the majority of the distributional characteristics of the data and eliminates the interference of outliers. The findings of the second regression analysis are provided in Column (4) of Table 4. The parameter estimates and significance levels do not change substantially from the baseline regression findings, with the coefficient of *ER* on health risk being −0.208 (*p* < 0.01), confirming the validity of the baseline regression results.

#### 3.3.5 Excluding the interference of the COVID-19 pandemic

The COVID-19 pandemic has posed a global public health emergency with far-reaching economic, social, and environmental impacts for all countries. During the COVID-19 outbreak, China adopted a sequestration strategy, which led to changes in PM_2.5_ concentrations. Concurrently, respiratory infection mortality increased surged. These factors influenced the health risk index we computed and confounded the results of the general analysis for the health benefits of environmental regulation. Therefore, to eliminate the potential influence of the COVID-19 pandemic on the empirical findings of this study, the observations for the years 2019–2020 were eliminated from the sample, and then a regression analysis was performed. As indicated in Column (5) of Table 4, with a coefficient of −0.123, *ER* is statistically significant at the 10% level, implying that an increase in the intensity of environmental regulation still significantly decreases health risks after controlling for potential data bias introduced by the COVID-19 pandemic.

### 3.4 Moderating effect of science and technology

According to the research findings, increasing the intensity of environmental regulation has a significant impact on lowering premature deaths due to the effect on PM_2.5_ and minimizing health risks. Based on this, with empirical evidence and theoretical modeling, the moderating role of science and technology in the relationship between environmental regulation and health risk will be examined in this section.

The regression results of the moderated effects model are displayed in Table 5. Specifically, the coefficient of the interaction term between the level of scientific and technological development and the intensity of environmental regulation is −8.723 with a statistically significant level of 10%, indicating that scientific and technological advancement enhances the health risk reduction effect of environmental regulation. A higher proportion of fiscal expenditure on scientific activities to fiscal revenue indicates that the area values scientific and technological innovation and is more capable of offering technical assistance and solutions for environmental preservation (50). Specifically, by promoting major technological innovations and transformative applications such as ecological product design, cleaner production processes, utilization of industrial linkages, and coordinated regional waste disposal and utilization, the advancement of science and technology facilitates the reduction of pollutants at the source and supports the efficient recycling and utilization of resources at multiple levels (51). In addition, real-time monitoring and analysis of air quality, pollution sources, and emissions can be achieved using scientific and technological means such as digital techniques, remote sensing techniques, and artificial intelligence, which provide data support for the formulation of scientific and reasonable environmental standards and policies and improve the accuracy and effectiveness of environmental regulation (52, 53). Additionally, these methods contribute to the early identification and punishment of unlawful emissions, thus strengthening environmental regulation, enforcement, and supervision (54). All these factors add to the mitigating effect of environmental regulation on health risks.

**Table 5:** Results of the moderating effect.

	(1)
	ED
*TE×ER*	−8.723^*^
	(−1.75)
*ER*	−0.025
	(−0.31)
*TE*	2.454^**^
	(2.32)
*RGDP*	−0.006
	(−0.83)
*LnPD*	0.332^*^
	(1.88)
*TIV*	−0.001^**^
	(−2.10)
*FSR*	−0.057^*^
	(−1.80)
*LnNH*	0.039^***^
	(4.47)
*LnAR*	−0.020
	(−0.31)
*LnAN*	−0.688^***^
	(−4.92)
*LnAP*	2.728
	(0.85)
*LnAT*	5.321^***^
	(3.99)
_cons	−21.287^***^
	(−2.81)
City FE	Yes
Time FE	Yes
*N*	3,035
*R* ^2^	0.993

t statistics in parentheses; ^*^p < 0.10, ^**^p < 0.05, ^***^p < 0.01.

### 3.5 Heterogeneity analysis

#### 3.5.1 Impacts of environmental regulation by region: coastal and inland cities

**Figure 3:**
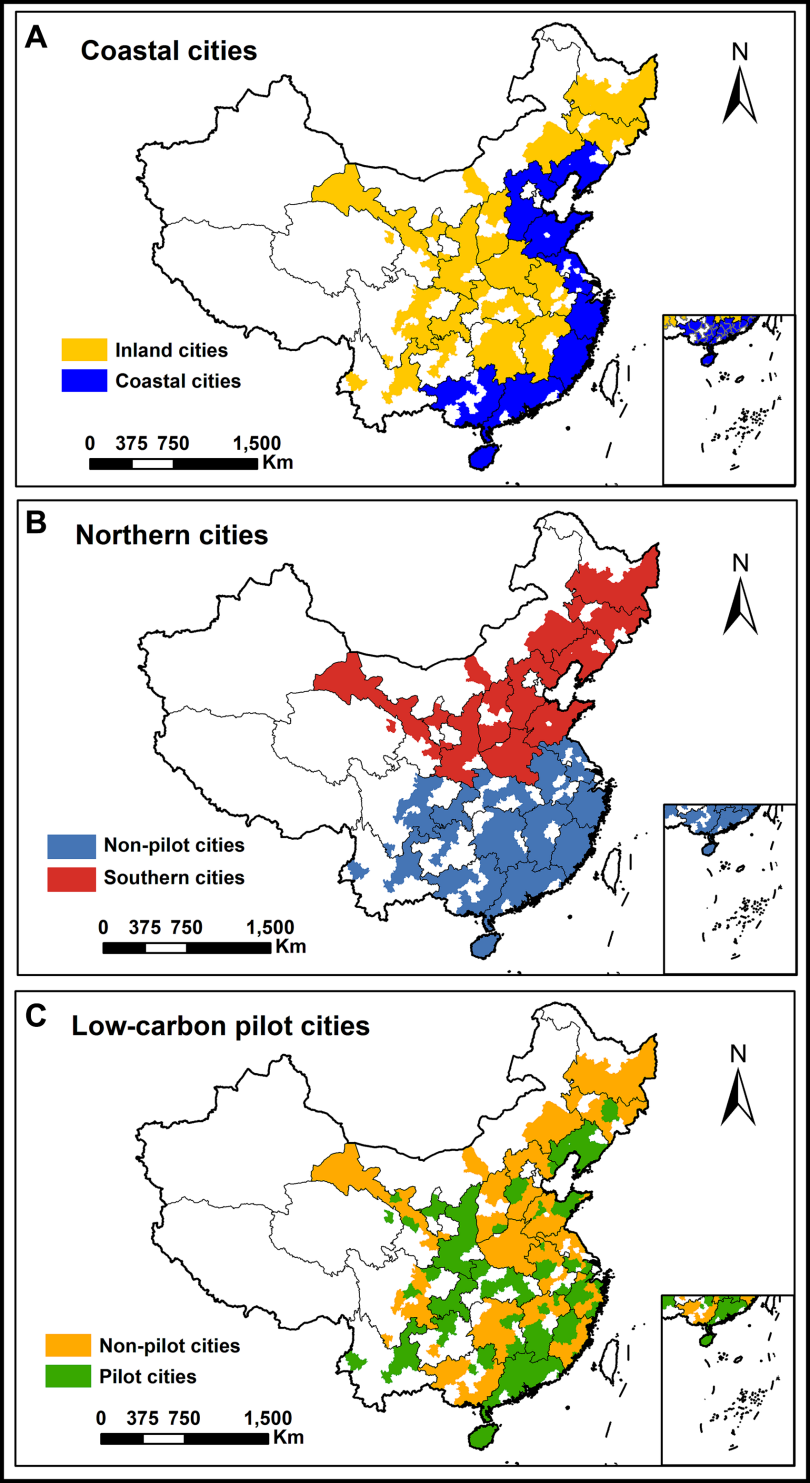
Spatial distribution by different characteristics. Northern and Southern cities **(A)**, coastal and inland cities **(B)**, low-carbon pilot and non-pilot cities **(C)**.

There are considerable disparities between coastal and inland cities in China in terms of economic structure, resource endowment, and degree of openness. These disparities potentially lead to varied feedback and adaptation in the face of environmental pressures (55, 56). For this reason, the sample cities in this research are divided into two groups based on whether they are coastal or inland cities, with group regressions used for comparative analyses, thus revealing the disparities between coastal and inland cities in terms of environmental regulation and health risks. Figure 3A illustrates the spatial distribution features of Chinese coastal and inland cities, and it is evident that coastal cities are mostly found in the eastern and southeastern regions of China.

Columns (1) and (2) of Table 6 display the effect of environmental regulation on health risk in inland and coastal cities, respectively. In particular, environmental regulation significantly decreases health risks in inland cities with a coefficient of-0.181 (*p* < 0.05), while the effect for coastal cities is not pronounced (*p* > 0.1). This is related to the special geographical environment and economic structure of coastal cities (57). First, the industrial structure of coastal cities is more diversified, covering a variety of high-energy-consuming and high-emission industries, such as iron and steel, chemical industry, and paper manufacturing. Secondly, well-developed transportation in coastal cities has also boosted pollution sources such as vehicle exhaust and ship emissions. Thirdly, complex meteorological conditions, such as sea breeze, sea fog, typhoons, also make air management in coastal cities difficult. These factors can affect the diffusion and removal of air pollutants, thus impeding the improvement of air quality. At the same time, inland cities have higher pollutant emissions with worse fine particle contamination than coastal cities (58), making the effects of environmental regulation more pronounced in inland cities in terms of lowering pollutant emissions, including PM_2.5_, and thereby mitigating health concerns.

**Table 6:** Results of the heterogeneity analysis.

	(1)	(2)	(3)	(4)	(5)	(6)
	Inland cities	Costal cities	Northern cities	Southern cities	Non-pilot cities	Pilot cities
*ER*	−0.181^**^	−0.018	−0.064	−0.209^**^	−0.228^*^	−0.075
	(−2.06)	(−0.23)	(−0.91)	(−2.23)	(−1.82)	(−1.26)
*RGDP*	−0.007	−0.002	0.020^***^	−0.026^*^	−0.014	−0.002
	(−0.22)	(−0.69)	(2.90)	(−1.89)	(−0.49)	(−1.10)
*LnPD*	0.189	0.582^***^	0.771^***^	−0.033	0.091	0.735^***^
	(0.72)	(4.06)	(8.13)	(−0.11)	(0.34)	(8.02)
*TIV*	−0.001^*^	0.000	−0.001	−0.000	−0.002^*^	−0.001
	(−1.66)	(0.00)	(−1.59)	(−0.10)	(−1.77)	(−1.59)
*FSR*	−0.047	0.032	0.008	−0.118^**^	−0.019	−0.042
	(−1.14)	(1.17)	(0.24)	(−2.44)	(−0.41)	(−1.60)
*LnNH*	0.047^***^	0.025^***^	0.021^***^	0.069^***^	0.051^***^	0.024^***^
	(3.77)	(3.66)	(4.27)	(3.56)	(3.81)	(3.81)
*LnAR*	−0.087	−0.063	0.051	−0.045	−0.150	0.079
	(−0.67)	(−0.96)	(0.60)	(−0.45)	(−1.22)	(1.03)
*LnAN*	−0.827^***^	−0.429^***^	−0.308^**^	−0.918^***^	−0.650^***^	−0.791^***^
	(−4.03)	(−3.12)	(−2.16)	(−4.96)	(−3.22)	(−4.70)
*LnAP*	−3.349	1.160	−12.341^*^	8.558^**^	−3.370	6.269^*^
	(−0.50)	(0.33)	(−1.95)	(2.37)	(−0.55)	(1.97)
*LnAT*	6.284^***^	0.546	8.007^***^	8.468^***^	5.541^***^	4.572^**^
	(2.91)	(0.35)	(4.72)	(2.62)	(3.09)	(2.61)
_cons	−25.688^**^	4.252	−39.085^***^	−36.931^**^	−20.786^**^	−19.600^**^
	(−2.15)	(0.47)	(−4.17)	(−2.09)	(−2.07)	(−1.99)
City FE	Yes	Yes	Yes	Yes	Yes	Yes
Time FE	Yes	Yes	Yes	Yes	Yes	Yes
*N*	1793	1,242	1,367	1,668	1718	1,317
*R* ^2^	0.992	0.997	0.997	0.990	0.991	0.997

t statistics in parentheses; ^*^p < 0.10, ^**^p < 0.05, ^***^p < 0.01.

#### 3.5.2 Impacts of environmental regulation by region: northern and southern cities

There are nonnegligible differences in climate, environment, and lifestyle between cities in southern and northern China. Northern cities, for example, burn large amounts of coal for heating in the winter, which produces harmful gasses in the combustion process, leading to increased air pollution and smog (59), whereas cities in the south have warmer temperatures in the winter without the need for heating but still face problems such as high humidity and poor air quality (60). In light of this, we investigated the disparities in the influence of environmental regulations on health risks in southern and northern cities. Figure 3B depicts the geographical arrangement of southern and northern cities, with the Qinling-Huaihe River serving as the dividing line.

Table 6 shows the influence of environmental regulation on health risk in southern and northern cities. Columns (3) and (4) show that the higher the intensity of environmental regulation is, the lower the health risk in southern cities, with a statistically significant coefficient of −0.209 (*p* < 0.05), although this impact is not significant (*p* > 0.1) in northern cities. The explanation for this discrepancy may lie in the substantial heating demand in northern cities during the winter, which leads to a multitude of dispersed pollution sources that are challenging to manage and mitigate effectively. Despite enormous expenditure, the government’s environmental regulatory efforts have failed to produce the expected environmental return on winter air pollution in northern cities due to factors such as over-reliance on government and local financial resources in the treatment process (38). In contrast, southern cities predominantly witness a concentration of pollutants within sectors such as industry and transportation (61), which are subject to more stringent environmental regulations, and as a result, these regulations have greater efficacy in reducing health-related risks.

#### 3.5.3 Impacts of environmental regulation by environmental characteristics

An arrangement for the environmental regulation of low-carbon pilot cities has been introduced in China to encourage low-carbon urban development and social reforms. This arrangement facilitates the achievement of climate goals. The implementation of the low-carbon pilot city policy has, however, led to variations in levels of pollution and environmental protection between pilot and nonpilot cities, implying that the health implications of environmental regulation may vary by city. Consequently, the sample cities in this study are separated into two categories, low-carbon pilot cities and nonpilot cities, for further examination. The spatial arrangement of low-carbon pilot and nonpilot cities is depicted in Figure 3C. As is evident from the geographical distribution, the pioneering low-carbon pilot cities have been strategically selected across several diverse provinces, encompassing both the coastal and inland areas, as well as four municipalities, covering most of the geographical area of China.

In Table 6, Columns (5) and (6) present regression results for non-low-carbon pilot cities and pilot cities, respectively. Notably, environmental regulation demonstrates a robust alleviating impact on health risk in non-low-carbon pilot cities, indicated by a coefficient of-0.228 at a significant level (*p* < 0.1). However, in the realm of low-carbon cities, the influence of environmental regulation fails to attain statistical significance. This divergence suggests that the health advantages of stringent environmental regulation are less pronounced in low-carbon pilot cities. To understand this phenomenon, one must consider the transformation of air quality. Low-carbon pilot cities have undeniably made great strides in enhancing their air quality through the proactive implementation of the low-carbon pilot program (62). As a result, the once-pervasive air pollution concerns have been noticeably mitigated, gradually fading into the now cleaner skies of these environmentally conscious cities. In contrast, non-low-carbon pilot cities still wrestle with the pressing issue of air pollution, making increased regulatory intensity more beneficial for them.

## 4 Discussion

Atmospheric pollution is a serious global concern, posing threats to both nature and human well-being. In response, governments have taken active measures to curb pollutant emissions and alleviate environmental damage. These measures offer more than just environmental protection, but also yield substantial health benefits, such as lower disease rates, increased life expectancy, and improved overall life quality. Consequently, appraising the health merits of environmental regulation is vital for discerning its ramifications on social welfare, enhancing cost–benefit analysis, and informing environmental policy alternatives. However, previous environmental regulation research has predominantly focused on its impacts on air pollution, greenhouse gas emissions, and energy consumption, with limited empirical studies on the implications of environmental regulation for health outcomes. Therefore, using the combined number of premature deaths from STK, IHD, and LC induced by outdoor PM_2.5_ exposure as a proxy for health risk, this study delves into the effect of environmental regulations on health risk, employing panel data from 276 Chinese cities over a period spanning from 2009 to 2020 to explore effective paths that reduce health risk. The results reveal that enhancing the intensity of environmental regulation significantly reduces health risks in cities, a finding that remains valid after multiple robustness tests, which demonstrates the health advantages of environmental regulation. Atmospheric pollution is a serious environmental issue that endangers human health by allowing harmful elements to enter the body through inhalation, causing irreversible damage. Air pollution has been proven to cause greater health risks than expected. Fine particulate matter (PM_2.5_), for example, with a diameter of less than 2.5 micrometers, can readily infiltrate the respiratory system and infect the lungs and bloodstream, posing a serious threat to the human body (63). Long-term exposure to high levels of PM_2.5_ can weaken people’s immunity and lead to chronic symptoms, such as coughing, breathlessness, migraines, and lung failure (3–5). Consequently, it is imperative to strengthen environmental regulation to reduce air pollution and protect human health. Compared with other studies that are merely theoretical, this paper quantitatively analyzes and proves the health benefits of environmental regulation by using high-precision long panel data and empirical studies, providing stronger evidence and support for proactive responses to air pollution and reducing health risks. Moreover, the policy consequences of environmental regulation are not static, but vary depending on factors such as regional location and environmental protection characteristics. This has often been overlooked in previous research on the health benefits of environmental regulation. Therefore, this paper examines not only the average impact of environmental regulation on health risks, but also the differential effect of environmental regulation on health consequences in terms of regional location and environmental protection characteristics. It also confirms the importance of scientific and technology levels in the process of environmental regulation exerting its effects, i.e., the higher the level of science and technology, the more significant the health influence of environmental regulation.

This paper investigates the impact of environmental regulations on the health risks associated with PM_2.5_ exposure. Nevertheless, our analysis is subject to several limitations. Firstly, we disregard the health consequences of other air pollutants, such as O_3_, which is a major contributor of respiratory and cardiovascular diseases. Therefore, future studies should examine the synergistic effects of multiple pollutants and the heterogeneity of different regions and populations in an integrated manner, to assess the implications of environmental regulation on health risks more accurately. Secondly, this paper only focuses on the health impacts of outdoor air quality, neglecting the effects of indoor air quality, which is also a crucial factor affecting residents’ health (63), particularly in China during the winter, where indoor pollution from activities such as coal combustion, cooking, and smoking elevates the risk of lung cancer, chronic obstructive pulmonary disease, and other diseases. To perform more thorough and comprehensive assessments of the association between environmental pollution and health risks, future studies should incorporate more diversified and accurate data, such as indoor and outdoor air quality monitoring data, as well as data on residents’ health status and behavior.

## 5 Conclusions and policy recommendations

This study provides evidence that an increasing intensity of environmental regulation can be associated with a reduction in health risks, with a 1-unit increase in the intensity of environmental regulation lowering the total number of local premature deaths from STK, IHD, and LC diseases by approximately 15.4%, a finding that holds up after multiple robustness tests. Additionally, the study highlights the positive synergy between scientific and technological advancements and environmental regulation in improving public health. Moreover, we also underscore the variation in health benefits across cities, with inland, southern, and non-low-carbon pilot cities experiencing more pronounced health benefits from environmental regulation. This research illuminates a promising path toward healthier and more sustainable environments.

Based on the findings of this research, three policy recommendations are proposed here. First, the social welfare effects of environmental regulation policies have been confirmed. Therefore, to enhance air quality and diminish health risks for residents, environmental regulation should be further improved by investing more in environmental protection and taking stricter measures against pollution sources. Second, the fostering of scientific and technological innovation and the promotion of clean technologies should be prioritized, along with the encouragement of enterprises to adopt eco-friendly production methods and equipment. Moreover, pollutant treatment and abatement technologies should be advanced, which will ultimately improve the health risk reduction effect of environmental policies. Third, it is crucial to tailor environmental regulatory policies by developing diverse and adaptable measures to suit the unique characteristics and needs of different cities, thereby improving the relevance and effectiveness of policies and leading to optimal health benefits for cities with varying sizes and environmental challenges.

## Data availability statement

Publicly available datasets were analyzed in this study. This data can be found at: https://weijing-rs.github.io/product.html and https://www.ecmwf.int/en/forecasts/dataset/ecmwf-reanalysis-v5.

## Ethics statement

Ethical approval was not required for the study involving humans in accordance with the local legislation and institutional requirements. Written informed consent to participate in this study was not required from the participants or the participants’ legal guardians/next of kin in accordance with the national legislation and the institutional requirements.

## Author contributions

QX: Data curation, Visualization, Writing – original draft. LW: Conceptualization, Data curation, Methodology, Writing – original draft. HH: Visualization, Writing – review & editing. ZH: Data curation, Resources, Writing – review & editing. WX: Conceptualization, Supervision, Writing – review & editing.

## Footnotes

^1^ https://weijing-rs.github.io/product.html

## References

[1] Xu B Chen J. How to achieve a low-carbon transition in the heavy industry? A nonlinear perspective. Renew Sust Energ Rev. (2021) 140:110708. doi: 10.1016/j.rser.2021.110708

[2] Liu K Lin B. Research on influencing factors of environmental pollution in China: a spatial econometric analysis. J Clean Prod. (2019) 206:356–64. doi: 10.1016/j.jclepro.2018.09.194

[3] Chen Z Liu P Xia X Wang L Li X. The underlying mechanism of PM_2.5_-induced ischemic stroke. Environ Pollut. (2022) 310:119827. doi: 10.1016/j.envpol.2022.119827, PMID: 35917837

[4] Raaschou-Nielsen O Antonsen S Agerbo E Hvidtfeldt UA Geels C Frohn LM. PM_2.5_ air pollution components and mortality in Denmark. Environ Int. (2023) 171:107685. doi: 10.1016/j.envint.2022.107685 36502699

[5] Hayes RB Lim C Zhang Y Cromar K Shao Y Reynolds HR. PM_2.5_ air pollution and cause-specific cardiovascular disease mortality. Int J Epidemiol. (2020) 49:25–35. doi: 10.1093/ije/dyz114, PMID: 31289812 PMC7124502

[6] Fujii H Managi S Kaneko S. Decomposition analysis of air pollution abatement in China: empirical study for ten industrial sectors from 1998 to 2009. J Clean Prod. (2013) 59:22–31. doi: 10.1016/j.jclepro.2013.06.059

[7] Hicks C Dietmar R. Improving cleaner production through the application of environmental management tools in China. J Clean Prod. (2007) 15:395–408. doi: 10.1016/j.jclepro.2005.11.008

[8] Zhu J Wu S Xu J. The abatement effect of total emission control policy: evidence from China. Energy Econ. (2023) 126:106978. doi: 10.1016/j.eneco.2023.106978

[9] Kahn AE. The economics of regulation: Principles and institutions. Cambridge, MA: MIT Press (1988).

[10] Li B Wu S. Effects of local and civil environmental regulation on green total factor productivity in China: a spatial Durbin econometric analysis. J Clean Prod. (2017) 153:342–53. doi: 10.1016/j.jclepro.2016.10.042

[11] Wang Y Shen N. Environmental regulation and environmental productivity: the case of China. Renew Sust Energ Rev. (2016) 62:758–66. doi: 10.1016/j.rser.2016.05.048

[12] Cole MA Elliott RJ. Determining the trade–environment composition effect: the role of capital, labor and environmental regulations. J Environ Econ Manag. (2003) 46:363–83. doi: 10.1016/S0095-0696(03)00021-4

[13] Hou J Teo TS Zhou F Lim MK Chen H. Does industrial green transformation successfully facilitate a decrease in carbon intensity in China? An environmental regulation perspective. J Clean Prod. (2018) 184:1060–71. doi: 10.1016/j.jclepro.2018.02.311

[14] Yuan B Xiang Q. Environmental regulation, industrial innovation and green development of Chinese manufacturing: based on an extended CDM model. J Clean Prod. (2018) 176:895–908. doi: 10.1016/j.jclepro.2017.12.034

[15] Du W Li M. Assessing the impact of environmental regulation on pollution abatement and collaborative emissions reduction: Micro-evidence from Chinese industrial enterprises. Environ Impact Assess Rev. (2020) 82:106382. doi: 10.1016/j.eiar.2020.106382

[16] Feng T Du H Lin Z Zuo J. Spatial spillover effects of environmental regulations on air pollution: evidence from urban agglomerations in China. J Environ Manag. (2020) 272:110998. doi: 10.1016/j.jenvman.2020.110998, PMID: 32854900

[17] Zhang K Xu D Li S. The impact of environmental regulation on environmental pollution in China: an empirical study based on the synergistic effect of industrial agglomeration. Environ Sci Pollut Res. (2019) 26:25775–88. doi: 10.1007/s11356-019-05854-z, PMID: 31267389

[18] Yu Y Dai C Wei Y Ren H Zhou J. Air pollution prevention and control action plan substantially reduced PM_2.5_ concentration in China. Energy Econ. (2022) 113:106206. doi: 10.1016/j.eneco.2022.106206

[19] Liu X Li Y Chen X Liu J. Evaluation of low carbon city pilot policy effect on carbon abatement in China: an empirical evidence based on time-varying DID model. Cities. (2022) 123:103582. doi: 10.1016/j.cities.2022.103582

[20] Hu X Sun Y Liu J Meng J Wang X Yang H. The impact of environmental protection tax on sectoral and spatial distribution of air pollution emissions in China. Environ Res Lett. (2019) 14:054013. doi: 10.1088/1748-9326/ab1965

[21] Tanaka S. Environmental regulations on air pollution in China and their impact on infant mortality. J Health Econ. (2015) 42:90–103. doi: 10.1016/j.jhealeco.2015.02.004, PMID: 25868145

[22] Yang M Chou S-Y. The impact of environmental regulation on fetal health: evidence from the shutdown of a coal-fired power plant located upwind of New Jersey. J Environ Econ Manag. (2018) 90:269–93. doi: 10.1016/j.jeem.2018.05.005

[23] Xu J Wang Y Liu W. Green to health: the impact of environmental regulation on health status. Sustain Cities Soc. (2023) 98:104839. doi: 10.1016/j.scs.2023.104839

[24] Zhou G Liu W Wang T Luo W Zhang L. Be regulated before be innovative? How environmental regulation makes enterprises technological innovation do better for public health. J Clean Prod. (2021) 303:126965. doi: 10.1016/j.jclepro.2021.126965

[25] Zhang Z Zhang G Li L. The spatial impact of atmospheric environmental policy on public health based on the mediation effect of air pollution in China. Environ Sci Pollut Res. (2022) 30:116584–600. doi: 10.1007/s11356-022-21501-6, PMID: 35779217

[26] Song Y Wei Y Zhu J Liu J Zhang M. Environmental regulation and economic growth: a new perspective based on technical level and healthy human capital. J Clean Prod. (2021) 318:128520. doi: 10.1016/j.jclepro.2021.128520

[27] Dai J Lv P Ma Z Bi J Wen T. Environmental risk and housing price: an empirical study of Nanjing, China. J Cleaner Prod. (2020) 252:119828. doi: 10.1016/j.jclepro.2019.119828

[28] Burnett RT Pope CA Ezzati M Olives C Lim SS Mehta S. An integrated risk function for estimating the global burden of disease attributable to ambient fine particulate matter exposure. Environ Health Perspect. (2014) 122:397–403. doi: 10.1289/ehp.1307049, PMID: 24518036 PMC3984213

[29] Lee CJ Martin RV Henze DK Brauer M Cohen A Donkelaar AV. Response of global particulate-matter-related mortality to changes in local precursor emissions. Environ Sci Technol. (2015) 49:4335–44. doi: 10.1021/acs.est.5b00873 25730303

[30] Wei J. Reconstructing 1-km-resolution high-quality PM_2.5_ data records from 2000 to 2018 in China: spatiotemporal variations and policy implications. Remote Sens Environ. (2021) 252:112136. doi: 10.1016/j.rse.2020.112136

[31] Wei J Li Z Cribb M Huang W Xue W Sun L. Improved 1 km resolution PM_2.5_ estimates across China using enhanced space–time extremely randomized trees. Atmos. Chem Phys. (2020) 20:3273–89. doi: 10.5194/acp-20-3273-2020

[32] Li J Zhu Y Kelly JT Jang CJ Wang S Hanna A. Health benefit assessment of PM_2.5_ reduction in Pearl River Delta region of China using a model-monitor data fusion approach. J Environ Manag. (2019) 233:489–98. doi: 10.1016/j.jenvman.2018.12.060, PMID: 30594114 PMC7260885

[33] Du K Cheng Y Yao X. Environmental regulation, green technology innovation, and industrial structure upgrading: the road to the green transformation of Chinese cities. Energy Econ. (2021) 98:105247. doi: 10.1016/j.eneco.2021.105247

[34] National Bureau of Statistics (NBS). Explanation of key statistical indicators. Available at: http://www.stats.gov.cn/zt_18555/ztsj/hjtjzl/2010/202303/t20230302_1921607.html (Accessed July 10, 2023).

[35] Hao Y Liu S Lu Z-N Huang J Zhao M. The impact of environmental pollution on public health expenditure: dynamic panel analysis based on Chinese provincial data. Environ Sci Pollut Res. (2018) 25:18853–65. doi: 10.1007/s11356-018-2095-y, PMID: 29713982

[36] Gu H Cao Y Elahi E Jha SK. Human health damages related to air pollution in China. Environ Sci Pollut Res. (2019) 26:13115–25. doi: 10.1007/s11356-019-04708-y, PMID: 30900129

[37] Kim MJ. Air pollution, health, and avoidance behavior: evidence from South Korea. Environ Resour Econ. (2021) 79:63–91. doi: 10.1007/s10640-021-00553-1

[38] Fan M He G Zhou M. The winter choke: coal-fired heating, air pollution, and mortality in China. J Health Econ. (2020) 71:102316. doi: 10.1016/j.jhealeco.2020.102316, PMID: 32179329

[39] Di Napoli C Barnard C Prudhomme C Cloke HL Pappenberger F. ERA5-HEAT: a global gridded historical dataset of human thermal comfort indices from climate reanalysis. Geosci Data J. (2021) 8:2–10. doi: 10.1002/gdj3.102

[40] Zhao J Jiang Q Dong X Dong K Jiang H. How does industrial structure adjustment reduce CO2 emissions? Spatial and mediation effects analysis for China. Energy Econ. (2022) 105:105704. doi: 10.1016/j.eneco.2021.105704

[41] Zhao D Dou Y Tong L. Effect of fiscal decentralization and dual environmental regulation on green poverty reduction: the case of China. Resources Policy. (2022) 79:102990. doi: 10.1016/j.resourpol.2022.102990

[42] Guo Q Zhong J. The effect of urban innovation performance of smart city construction policies: evaluate by using a multiple period difference-in-differences model. Technol Forecast Soc Chang. (2022) 184:122003. doi: 10.1016/j.techfore.2022.122003

[43] Zeng H Zhang X Zhou Q Jin Y Cao J. Tightening of environmental regulations and corporate environmental irresponsibility: a quasi-natural experiment. Environ Dev Sustain. (2022) 24:13218–59. doi: 10.1007/s10668-021-01988-8

[44] Yang M Chen H Long R Yang J. How does government regulation shape residents’ green consumption behavior? A multi-agent simulation considering environmental values and social interaction. J Environ Manag. (2023) 331:117231. doi: 10.1016/j.jenvman.2023.117231, PMID: 36634421

[45] Fan M Yang P Li Q. Impact of environmental regulation on green total factor productivity: a new perspective of green technological innovation. Environ Sci Pollut Res. (2022) 29:53785–800. doi: 10.1007/s11356-022-19576-2, PMID: 35288859

[46] Rahman MM Alam K. Clean energy, population density, urbanization and environmental pollution nexus: evidence from Bangladesh. Renew Energy. (2021) 172:1063–72. doi: 10.1016/j.renene.2021.03.103

[47] Yitshak-Sade M Kloog I Novack V. Do air pollution and neighborhood greenness exposures improve the predicted cardiovascular risk? Environ Int. (2017) 107:147–53. doi: 10.1016/j.envint.2017.07.011, PMID: 28735151

[48] Stock JH, Testing for weak instruments in linear IV regression.

[49] Xue W Wang L Yang Z Xiong Z Li X Xu Q. Can clean heating effectively alleviate air pollution: an empirical study based on the plan for cleaner winter heating in northern China. Appl Energy. (2023) 351:121923. doi: 10.1016/j.apenergy.2023.121923

[50] Zhu Y Liu Z Feng S Lu N. The role of fiscal expenditure on science and technology in carbon reduction: evidence from provincial data in China. Environ Sci Pollut Res. (2022) 29:82030–44. doi: 10.1007/s11356-022-21500-7, PMID: 35748993

[51] Sun H Zhang Z Liu Z. Does air pollution collaborative governance promote green technology innovation? Evidence from China. Environ Sci Pollut Res. (2022) 29:51609–22. doi: 10.1007/s11356-022-19535-x, PMID: 35249197

[52] Mishra S Jena L Tripathy HK Gaber T. Prioritized and predictive intelligence of things enabled waste management model in smart and sustainable environment. PLoS One. (2022) 17:e0272383. doi: 10.1371/journal.pone.0272383, PMID: 35951589 PMC9371262

[53] Himeur Y Rimal B Tiwary A Amira A. Using artificial intelligence and data fusion for environmental monitoring: a review and future perspectives. Inform Fusion. (2022) 86-87:44–75. doi: 10.1016/j.inffus.2022.06.003

[54] Yang X Wu H Ren S Ran Q Zhang J. Does the development of the internet contribute to air pollution control in China? Mechanism discussion and empirical test. Struct Chang Econ Dyn. (2021) 56:207–24. doi: 10.1016/j.strueco.2020.12.001

[55] Zheng W Chu J Bambrick H Wang N Mengersen K Guo X. Impact of environmental factors on diabetes mortality: a comparison between inland and coastal areas. Sci Total Environ. (2023) 904:166335. doi: 10.1016/j.scitotenv.2023.166335, PMID: 37591381

[56] Zhu S He C Liu Y. Going green or going away: environmental regulation, economic geography and firms’ strategies in China’s pollution-intensive industries. Geoforum. (2014) 55:53–65. doi: 10.1016/j.geoforum.2014.05.004

[57] Nie X Mao H Li P Li T Zhou J Wu Y. Total gaseous mercury in a coastal city (Qingdao, China): influence of sea-land breeze and regional transport. Atmos Environ. (2020) 235:117633. doi: 10.1016/j.atmosenv.2020.117633

[58] Shu L Xie M Gao D Wang T Fang D Liu Q. Regional severe particle pollution and its association with synoptic weather patterns in the Yangtze River Delta region, China. Atmos Chem Phys. (2017) 17:12871–91. doi: 10.5194/acp-17-12871-2017

[59] Zhang Z Wang W Cheng M Liu S Xu J He Y. The contribution of residential coal combustion to PM_2.5_ pollution over China’s Beijing-Tianjin-Hebei region in winter. Atmos Environ. (2017) 159:147–61. doi: 10.1016/j.atmosenv.2017.03.054

[60] Li F Zhou T. Effects of urban form on air quality in China: an analysis based on the spatial autoregressive model. Cities. (2019) 89:130–40. doi: 10.1016/j.cities.2019.01.025

[61] Zou B-B Huang X-F Zhang B Dai J Zeng L-W Feng N. Source apportionment of PM_2.5_ pollution in an industrial city in southern China. Atmospheric. Pollut Res. (2017) 8:1193–202. doi: 10.1016/j.apr.2017.05.001

[62] Chen J Luo W Ren X Liu T. The local-neighborhood effects of low-carbon city pilots program on PM_2.5_ in China: a spatial difference-in-differences analysis. Sci Total Environ. (2023) 857:159511. doi: 10.1016/j.scitotenv.2022.159511, PMID: 36283527

[63] Kumar P Singh AB Arora T Singh S Singh R. Critical review on emerging health effects associated with the indoor air quality and its sustainable management. Sci Total Environ. (2023) 872:162163. doi: 10.1016/j.scitotenv.2023.162163, PMID: 36781134

